# Maternal mental health and nutritional status of six-month-old infants

**DOI:** 10.1590/S1518-8787.2016050006237

**Published:** 2016-03-10

**Authors:** Bruna Kulik Hassan, Guilherme Loureiro Werneck, Maria Helena Hasselmann

**Affiliations:** I Programa de Pós-Graduação em Saúde Coletiva. Instituto de Medicina Social. Universidade do Estado do Rio de Janeiro. Rio de Janeiro, RJ, Brasil; II Departamento de Epidemiologia. Instituto de Medicina Social. Universidade do Estado do Rio de Janeiro. Rio de Janeiro, RJ, Brasil; III Departamento de Nutrição Social. Instituto de Nutrição. Universidade do Estado do Rio de Janeiro. Rio de Janeiro, RJ, Brasil

**Keywords:** Maternal Welfare, psychology, Mental Health, Postpartum Period, Depression, Postpartum, Infant Nutrition, Mother-Child Relations, Cross-Sectional Studies

## Abstract

**OBJECTIVE:**

To analyze if maternal mental health is associated with infant nutritional status at six month of age.

**METHODS:**

A cross-sectional study with 228 six-month-old infants who used primary health care units of the city of Rio de Janeiro, Southeastern Brazil. Mean weight-for-length and mean weight-for-age were expressed in z-scores considering the 2006 World Health Organization reference curves. Maternal mental health was measured by the 12-item General Health Questionnaire. The following cutoff points were used: ≥ 3 for common mental disorders, ≥ 5 for more severe mental disorders, and ≥ 9 for depression. The statistical analysis employed adjusted linear regression models.

**RESULTS:**

The prevalence of common mental disorders, more severe mental disorders and depression was 39.9%, 23.7%, and 8.3%, respectively. Children of women with more severe mental disorders had, on average, a weight-for-length 0.37 z-scores lower than children of women without this health harm (p = 0.026). We also observed that the weight-for-length indicator of children of depressed mothers was, on average, 0.67 z-scores lower than that of children of nondepressed women (p = 0.010). Maternal depression was associated with lower mean values of weight-for-age z-scores (p = 0.041).

**CONCLUSIONS:**

Maternal mental health is positively related to the inadequacy of the nutritional status of infants at six months.

## INTRODUCTION

Child malnutrition persists as a major public health problem in developing countries, contributing to 1/3 of under-five deaths^28^. According to estimates from six cohort studies in developing countries, its eradication could prevent a million deaths from pneumonia, 800 thousand from diarrhoea, 500 thousand from malaria and 200 thousand from measles^2^.

According to joint data from the United Nations Children’s Fund (UNICEF), the World Bank, and the World Health Organization (WHO), 161 million children under the age of five were stunted in 2013, which is equivalent to 25.0% of the world population^23^. Although Brazil presents a better current situation regarding child malnutrition, with 6.0% of children under five years of age stunted, the distribution of this indicator is uneven, with a higher prevalence in the North (8.5%), in children under one year of age (8.8%) and among those who live in families with a monthly *per capita* income of up to 1/4 of the minimum wage (8.2%)^10^.

There is extensive literature on determinants of child growth. The most important are social, economic and environmental factors such as parents’ educational attainment and housing conditions, maternal age and work, family size, birth weight, recurrent infections and dietary habits^16^ ^,^ ^30^.

Such evidence does not solve, however, the issue of inadequate child growth determination. Even after the socioeconomic and demographic changes and improvement in the care provided by health services in the last decades, there are still children with nutritional deficits in developing countries^28^ ^,^ ^29^. In addition, family members under the same conditions of life and living in the same house have different nutritional status^11^.

In this regard, investigations have focused on the contribution of psychosocial aspects in the process, pointing to alcoholism and lack of social support, as well as aspects related to motherhood, such as factors associated with nutritional deficits in childhood^1^ ^,^ ^26^. Some studies suggest that factors present within the family environment could negatively affect the maternal capacity to provide adequate care for the child, contributing to child malnutrition^1^ ^,^ ^26^.

Although increasing, the literature addressing the impact of maternal mental health on children’s nutritional status has contradictory results. While some studies showed a worse growth pattern in children whose mothers presented some psychological disorder when compared with the children of women without disorders, other investigations conducted in developing countries did not show significant differences for this relation^6^ ^,^ ^18^ ^,^ ^19^ ^,^ ^22^. To clarify this issue, the objective of the present study was to examine whether maternal mental health is associated with child nutritional status in the sixth month of life.

## METHODS

This cross-sectional study is part of a prospective cohort whose central objective is to investigate the social determinants of the growth of children in the first year of life.

Its population is composed of newborns who used four primary health care units of the municipality of Rio de Janeiro, Southeastern Brazil, at the time of vaccination (BCG) or Guthrie test, referred directly from the maternity hospitals where they were born, as recommended by the Rio de Janeiro Municipal Health Department (SMS-RJ) program “*Acolhimento mãe-bebê*” (Mother-baby welcoming). Primary health care units were chosen intentionally, considering the average number of newborns registered in the program every month and the geographical variability, to represent different population groups.

The information relating to the first interview (first month) and to the one six months after birth were included in this study. Excluding those with congenital anomalies, cerebral palsy and six twins, 228 children composed the sample. Throughout data collection, between June 2005 and June 2008, the principal investigator of the research supervised the interviewers. Questionnaires with closed, precoded questions, and other open or semi-open questions were applied in face-to-face interviews with the children’s mothers.

To obtain the outcome variables reflecting the child’s nutritional status, the weight and length of children at six months of age were measured without any clothing or accessories. Weight was measured using a Filizola pediatric scale with a maximum capacity of 15 kg (0.1 kg resolution). Length was measured using a wooden horizontal infant stadiometer specially constructed for the study (1 mm resolution). The anthropometric training consisted of the following steps: (a) introduction on nutritional assessment of children under two years of age; (b) theoretical presentation of the techniques for weighing and measuring children under two years of age; and (c) standardization of anthropometric measurements, a procedure recommended by Habicht^5^ (1974). Based on these measurements, we estimated the variables weight-for-length and weight-for-age according to sex, expressed as z-scores, using the new growth chart for children under five^31^.

To assess maternal mental health six months after giving birth, we used the Brazilian Portuguese adapted and validated version of the 12-item General Health Questionnaire (GHQ-12)^12^. This instrument uses the two weeks before filling the questionnaire as reference period and reflects feelings of depression and anxiety, inability to cope with usual situations and lack of confidence^4^.

Each item of GHQ-12 contains four response options, the first two considering the recent experience of the participant in relation to a particular symptom or behavior as absent (0 points assigned) and the last two as present (1 point assigned). Thus, summing the points of each item generates a scale from zero to 12 possible points. Three variables were established to assess maternal mental health with this instrument. The variable common mental disorders (CMD) was set from the cut-off point of at least three positive responses. This cut-off point shows good sensitivity (85.0%), without any significant loss of specificity (79.0%)^12^. The variable more severe mental disorders was identified by the cut-off point of at least five positive responses. Villano^a^ found a 76.0% sensitivity and a 59.0% specificity for this cut-off point in an outpatient clinic of a university hospital in Rio de Janeiro. Finally, for the variable depression we used the cut-off point of at least nine positive items, as in the study by Ozdemir and Remaki^17^, who found a 75.0% sensitivity and a 77.0% specificity.

The covariables investigated were: maternal age (< or ≥ 20 years), maternal work (yes or no), prematurity (yes or no), birth weight (in grams), number of children under five years of age, mother’s educational attainment (full years of schooling) and social network. To evaluate the social network, participants were asked “how many friends do you feel comfortable with and can talk about almost anything?” and “how many relatives do you feel comfortable with and can talk about almost anything?”. We subsequently added the number of relatives and friends mentioned to compose this variable.

Initially, we estimated the mean z-scores for outcomes along with the prevalence of exposure variables as well as sociodemographic, child, and maternal characteristics. Crude analyses used simple linear regression (categorical covariables and central exposure variables) and Spearman’s correlation test (numerical covariables). In multivariate analysis, linear regression models were adjusted by the covariables presenting associations of p ≤ 0.20 in the crude analyses.

Three methods of diagnosis of the regression models were used to evaluate the quality of fit: Q-Q plot, studentized residuals *versus* predicted values graphs, and Cook’s distance. All multivariate models showed good fit and only one outlier was found, but maintained in the analysis.

Data were stored in the application EpiInfo version 6.04 and analyzed in statistical packages R version 2.9.0 and Stata version 9.2. The software WHO Anthro version 3.0.1 was used to estimate the indexes weight-for-length and weight-for-age.

The study was approved by the Ethics Committee of the Instituto de Medicina Social of the Universidade do Estado do Rio de Janeiro. Mothers needed to sign an informed consent form to participate in the study.

## RESULTS

Table 1 presents the characteristics of the study population. The children’s mean age was 6.46 months, and 52.4% of the sample were boys. The minority of the children were premature and had low birth weight. For the indexes weight-for-length and weight-for-age, the mean z-scores were 0.23 and 0.05, respectively.

**Table 1:** Characteristics of the study population. Rio de Janeiro, RJ, Southeastern Brazil, 2005-2008.

Categorical variables	n*	%
Sex		
Male	122	52.4
Female	111	47.2
Birth weight (grams)		
Low (< 2,500)	16	6.9
Adequate (≥ 2,500)	216	93.1
Prematurity		
No	199	90.9
Yes	20	9.1
Maternal work		
No	157	71.7
Yes	62	28.3
Maternal age (years)		
≥ 20	173	77.2
< 20	51	22.8
CMD (GHQ-12)		
No (< 3)	137	60.1
Yes (≥ 3)	91	39.9
More severe disorders (GHQ-12)		
No (< 5)	174	76.3
Yes (≥ 5)	54	23.7
Depression (GHQ-12)		
No (< 9)	209	91.7
Yes (≥ 9)	19	8.3

Numeric variables	Mean	SD

Weight-for-length	0.23	1.08
Weight-for-age	0.05	1.07
Birth weight (grams)	3.21	0.48
Child’s age (months)	6.46	0.80
Maternal age (years)	25.5	6.57
Maternal education (years of schooling)	8.49	2.81
Number of children < 5 years of age	1.24	0.46
Social network (friends and relatives)	3.67	2.87

* Totals may vary due to missing values in some variables.

Mother’s mean age was 25.5 years. They also had, on average, 8.5 years of schooling, 1.24 children and 3.67 friends or relatives who they could trust to tell almost everything (social network). There was a high percentage of adult women who were not working at the time of the interview. Approximately 40.0% of mothers were detected with CMD, 24.0% with more severe mental disorders, and 8.0 percent with depression.

In the crude analysis (Tables 2 and 3), only birth weight was significantly associated with weight-for-length and weight-for-age, and was the variable selected to be included in the multiple linear regression models.

The crude and adjusted associations between exposure variables and children’s nutritional status are shown in Table 4. More severe mental disorders and depression were associated with lower average weight-for-length z-scores (Table 4). Children of women with more severe mental disorders had, on average, a weight-for-length 0.37 z-scores lower than children of women without this health condition (p = 0.026). Children of women with depression had, on average, 0.67 z-scores lower than children of nondepressed mothers (p = 0.010). We observed no statistically significant association between CMD and weight-for-length.

**Table 2:** Bivariate analysis between weight-for-length, weight-for-age, and categorical covariables (simple linear regression). Rio de Janeiro, RJ, Southeastern Brazil, 2005-2008.

Variable	Weight-for-length			Weight-for-age		
	
	Mean	SD	p	Mean	SD	p
Prematurity						
No	0.21	1.09	-	0.05	1.06	-
Yes	0.18	1.02	0.906	-0.17	1.14	0.397
Maternal work						
No	0.23	1.06	-	0.04	1.05	-
Yes	0.28	1.19	0.797	0.07	1.15	0.860
Maternal age (years)						
≥ 20	0.21	1.09	-	0.06	1.07	-
< 20	0.26	1.08	0.766	-0.003	1.05	0.731

**Table 3:** Spearman’s correlation coefficients between the indexes weight-for-length and weight-for-age and the numerical covariables. Rio de Janeiro, RJ, Southeastern Brazil, 2005-2008.

Variable	Weight-for-length		Weight-for-age	
	
	Coefficient	p	Coefficient	p
Birth weight (grams)	0.225	< 0.001	0.484	< 0.001
Maternal education (years of schooling)	-0.046	0.485	-0.010	0.875
Number of children < 5 years of age	-0.001	0.990	-0.055	0.415
Social network (friends and relatives)	-0.052	0.428	0.002	0.970

**Table 4:** Crude and adjusted linear regression coefficients for the association between weight-for-length and weight-for-age with main exposure variables. Rio de Janeiro, RJ, Southeastern Brazil, 2005-2008.

Variable	Weight-for-length				Weight-for-age			
	
	Crude		Adjusted*		Crude		Adjusted*	
			
	Coefficient	p	Coefficient	p	Coefficient	p	Coefficient	p
CMD (GHQ-12 ≥ 3)	-0.080	0.585	-0.053	0.714	0.022	0.882	0.048	0.708
More serious disorders (GHQ-12 ≥ 5)	-0.393	0.019	-0.368	0.026	-0.025	0.884	-0.014	0.928
Depression (GHQ-12 ≥ 9)	-0.674	0.011	-0.667	0.010	-0.474	0.065	-0.463	0.041

* Model adjusted by the birth weight variable.

Children whose mothers were detected with depression showed, on average, weight-for-age 0.46 z-scores lower than children of women without depression (p = 0.041). The other exposure variables did not show any statistically significant association with this outcome (Table 4).

## DISCUSSION

The results of this study showed that maternal mental health is related to children’s nutritional status at six months of life. In the adjusted models we found an association between lower mean weight-for-length z-scores and exposure variables, except CMD. The mean weight-for-age z-score was only associated with maternal depression.

The literature confirms the findings of this study because it has indicated increased risk of child malnutrition in the first six months of life among children of women with postpartum depression^14^ ^,^ ^18^ ^,^ ^19^. A recent longitudinal study in Bangladesh found that depression between two and three months postpartum is a risk factor for underweight between six and eight months of age, and prenatal depression is for length-for-age deficit between two and three months and between six and eight months of age^14^.

A meta-analysis of 17 studies on maternal mental health and childhood growth has verified that there is a greater chance of weight- and length-for-age deficits among children of women with depression or depressive and anxious symptoms compared with children of nondepressed women^27^. The effect on these nutritional deficits was greater in the analyses using the diagnosis of depression^27^. The authors also found a greater effect in longitudinal studies, but the exposure investigated in them was to postpartum depression and the result for length-for-age deficit was not significative^27^.

In this study, we noted that the regression coefficient increases with the increase in the severity of exposure, i.e., the association is stronger between nutritional outcomes and more severe disorders and, therefore, depression. As the biological gradient, or dose-response effect, is a relevant criterion to infer causality, the findings of this study, although sectional in nature, reinforce the hypothesis of a causal effect between maternal mental health and child malnutrition. In this context, the exposure effect could only occur beyond certain threshold, which, in the case of this study, would be the presence of more severe mental disorders. Accordingly, the level of exposure of CMD would be below the exposure threshold, which could justify its lack of association with the outcome studied.

There is still no consensus in the literature on the association between maternal CMD and nutritional deficit. While some findings show a positive relationship between these events^6^ ^,^ ^15^ ^,^ ^21^ ^,^ ^22^, others show non-significant or null associations^6^ ^,^ ^13^ ^,^ ^15^ ^,^ ^25^. The presence of CMD in a single moment may be insufficient to modify the outcomes related to nutritional status.

A recent prospective cohort in Vietnam assessed CMD among mothers in the last trimester of pregnancy or between four and six weeks postpartum (baseline) and again at 15 months postpartum, along with length-for-age. According to the authors, CMD at baseline was not associated with linear growth, but there was a higher risk of maternal CMD at 15 months postpartum among women with perinatal CMD. The persistence of perinatal CMD and CMD at 15 months postpartum was inversely associated with length-for-age at 15 months^3^.

A case-control study conducted in Brazil found nearly twice the chance of weight-for-height deficit in children up to six years of age whose mothers presented CMD^22^. On the other hand, Stewart et al.^25^ found no association between the presence of CMD and lower postpartum mean z-scores for weight-for-age in children aged nine months. Medhin et al.^13^, in an Ethiopian cohort of more than a thousand participants, observed no effect of CMD in the last trimester of pregnancy, at two months postpartum, or its persistence between these two moments, on weight- or length-for-age at six and 12 months of age, not even in the continuous assessment of these nutritional indexes.

Part of the heterogeneity of results among studies can be explained by the different postpartum moments in which investigations were conducted or by the method of measurement of mental disorders in this phase. Additionally, differences in literature can occur due to different methodologies employed in the choice of the outcome variable or its classification, or due to the use of different anthropometric indicators. In relation to the classification of outcome variables, for example, some studies have used the cut-off point of -2 z-scores to detect nutritional deficit in children^6^ ^,^ ^15^ ^,^ ^22^ while others used percentiles^18^ or mean z-scores^21^ ^,^ ^25^. Given this, a comparison of the findings is limited.

The results of the studies also varied according to the indicator used to assess impaired child growth as well as the country in which the study was conducted^6^ ^,^ ^15^ ^,^ ^25^. Harpham et al.^6^ observed an association of CMD with length-for-age deficit in India, but not with weight-for-age deficit. The opposite occurred in Vietnam, with a statistically significant association with low weight-for-age and no association with length-for-age. The authors did not find significant results for Peru and Ethiopia. Similar results were found when Nguyen et al.^15^ assessed populations from Bangladesh, Vietnam and Ethiopia. According to some authors, the differences in results may be related to cultural differences in dietary practices and child care among the countries studied, as well as socioeconomic differences such as maternal educational attainment and food security in the house^6^ ^,^ ^22^ ^,^ ^27^.

The potential mechanisms to explain the relationship between maternal mental health and child growth are not clear yet. One hypothesis is that maternal mental health problems may impair the mother’s ability to provide proper care for the child, providing inadequate hygiene practices, insufficient food supply and lower bond with the child, which could compromise child growth^1^. Women with depressed mood are more prone to be irritable and hostile to their children and, under these circumstances, may neglect care when the baby is hungry or unable to suck breast milk properly. They may also neglect hygiene and sanitation in food preparation, which can lead to diarrhea and, consequently, to nutritional deficit^1^ ^,^ ^13^ ^,^ ^15^.

Postpartum depressive symptoms have been shown to represent a higher risk for premature interruption of breastfeeding, which reflects the mother’s inability to provide care in feeding^7^ ^,^ ^19^. The interruption of breastfeeding harms the evolution of weight gain^22^. Less diverse complementary feeding, energy intake, and frequency of meals have also been reported among children of women with depressive and anxious symptoms^8^ ^,^ ^15^.

Another potential cause of the relationship between maternal mental health and child malnutrition could be improper maternal health care, as disease prevention and search for care and health services when the child is sick, leading to illness and child malnutrition. Nguyen et al.^15^ showed that maternal CMD are strongly associated with diarrhea and acute respiratory infection.

Despite the hypothesis that mothers’ psychological problems may lead to nutritional status compromise in children, there is still no consensus about the temporality of this relationship. Compromised child growth may increase mothers’ concerns, promote feelings of inability to care for the child, and increase family pressures, inducing tension, anxiety, and even more serious depressive states^9^ ^,^ ^24^. Some studies observed that mothers of children with health problems, low birth weight and growth deficit had an increased risk of presenting more severe mental disorders or depression in the first year of the child’s life^9^ ^,^ ^20^ ^,^ ^27^.

The differences in literature strengthen the need for more longitudinal investigations in the first year after childbirth. It is also necessary to conduct investigations focusing on possible mechanisms that may interfere in these relationships.

Although GHQ-12 is widely used for CMD screening in literature, some studies have recently been using this instrument for more severe disorders and depression^17^ ^,^ ^20^. Zubaran et al.^32^ observed a significant linear correlation between GHQ-12 and scales widely used to assess postpartum depression (as the Postpartum care Depression Screening Scale [PDSS] and the Edinburgh Postnatal Depression Scale [EPDS]), recommending the use of general health questionnaires for this health condition.

In this perspective, this study contributes to the discussion on the determinants of child growth and confirms the hypothesis of an association between maternal mental health and nutritional deficits in infancy.

Finally, our results indicate the need for incorporation of mental health into health care policies and programs including prevention and treatment of mental disorders, aiming at improving child nutritional status. In particular, we recommend more careful attention in health services routine, both to mothers of children with nutritional deficits and to children of women with psychological disorders. The use of accessible instruments in health services to detect psychological problems after childbirth has been recommended^b^. A simple approach would be the inclusion of screening questionnaires as GHQ-12 and team training, aiming to increase the detection of this population in service routines.

## Footnotes

a Villano LA. Problemas psicológicos e morbidade psiquiátrica em serviços de saúde não-psiquiátricos: o ambulatório de clínica geral [these]. São Paulo (SP): Universidade Federal de São Paulo; 1998.

b World Health Organization, Department of Mental Health and Substance Abuse. Maternal mental health and child health and development in low and middle income countries: report of the meeting held in Geneva, Switzerland; 2008 30 Jan- Feb 2008. Geneva: World Health Organization; 2008.
