# Supplementary material for: Maternal mental health and nutritional status of six-month-old infants
Source: Rev Saude Publica. 2016 Mar 10;50:7. doi: 10.1590/S1518-8787.2016050006237 (PMC4794770; doi:10.1590/S1518-8787.2016050006237)
Supplement: Translation

# Translation: Saúde mental materna e estado nutricional de crianças aos seis meses de vida

**Authors:** Bruna Kulik Hassan, Guilherme Loureiro Werneck, Maria Helena Hasselmann

**Affiliations:** I Programa de Pós-Graduação em Saúde Coletiva. Instituto de Medicina Social. Universidade do Estado do Rio de Janeiro. Rio de Janeiro, RJ, Brasil; II Departamento de Epidemiologia. Instituto de Medicina Social. Universidade do Estado do Rio de Janeiro. Rio de Janeiro, RJ, Brasil; III Departamento de Nutrição Social. Instituto de Nutrição. Universidade do Estado do Rio de Janeiro. Rio de Janeiro, RJ, Brasil

**Keywords:** Bem-Estar Materno, psicologia, Saúde Mental, Período Pós-Parto, Depressão Pós-Parto, Nutrição do Lactente, Relações Mãe-Filho, Estudos Transversais

## Abstract

**OBJETIVO:**

Analisar se saúde mental materna associa-se ao estado nutricional infantil no sexto mês de vida.

**MÉTODOS:**

Estudo seccional com 228 crianças aos seis meses atendidas em unidades básicas de saúde do município do Rio de Janeiro. Médias de peso-para-comprimento e peso-para-idade foram expressas em escores z considerando as curvas de referência do World Health Organization 2006. A saúde mental materna foi aferida pelo *General Health Questionnaire* com 12 itens. Foram empregados os pontos de corte ≥ 3 para transtornos mentais comuns, ≥ 5 para transtornos mentais mais graves e ≥ 9 para depressão. Na análise estatística empregaram-se modelos de regressão linear ajustados.

**RESULTADOS:**

As prevalências de transtornos mentais comuns, transtornos mentais mais graves e depressão foram de 39,9%, 23,7% e 8,3%, respectivamente. Para peso-para-comprimento, filhos de mulheres com transtornos mentais mais graves tinham, em média, 0,37 escores z mais baixos do que filhos de mulheres sem este agravo (p = 0,026). Também para o indicador peso-para-comprimento observou-se que, em relação aos filhos de mulheres não deprimidas, crianças de mães deprimidas apresentaram, em média, 0,67 escores z mais baixos (p = 0,010). Depressão materna esteve associada com valores médios mais baixos de escore z de peso-para-idade (p = 0,041).

**CONCLUSÕES:**

A saúde mental materna está positivamente relacionada à inadequação do estado nutricional de crianças aos seis meses.

## INTRODUÇÃO

A desnutrição infantil persiste como importante problema de saúde pública em países em desenvolvimento, contribuindo para 1/3 das mortes em menores de cinco anos^28^. Segundo estimativas de seis estudos de coorte de países em desenvolvimento, sua erradicação poderia prevenir um milhão de mortes por pneumonia, 800 mil por diarreia, 500 mil por malária e 200 mil por sarampo^2^.

Segundo dados conjuntos da *United Nations Children’s Fund* (UNICEF), do *The World Bank* e da Organização Mundial de Saúde (OMS), 161 milhões de crianças menores de cinco anos apresentaram déficit de estatura em 2013, número equivalente a 25,0% da população mundial^23^. Ainda que o Brasil apresente melhor cenário atual de desnutrição infantil, com 6,0% das crianças menores de cinco anos com déficit de estatura, a distribuição deste indicador é desigual, com prevalências mais elevadas no Norte (8,5%), em menores de um ano (8,8%) e entre aqueles que vivem em famílias com rendimento mensal *per capita* de até 1/4 do salário mínimo (8,2%)^10^.

Existe vasta literatura acerca dos determinantes do crescimento infantil. Dentre eles, destacam-se fatores socioeconômicos e ambientais, tais como a escolaridade dos pais e condições de moradia, trabalho e idade materna, tamanho da família, peso da criança ao nascer, infecções recorrentes e práticas alimentares^16^ ^,^ ^30^.

Essas evidências não encerram, no entanto, a questão sobre a determinação do crescimento infantil inadequado. Isso porque, mesmo após as transformações socioeconômicas e demográficas e a melhoria na atenção prestada pelos serviços de saúde nas últimas décadas, ainda persistem crianças com déficits nutricionais nos países em desenvolvimento^28^ ^,^ ^29^. Ademais, famílias com as mesmas condições de vida e vivendo em um mesmo domicílio apresentam diferentes estados nutricionais^11^.

Nesse sentido, investigações têm focalizado a contribuição dos aspectos psicossociais neste processo, apontando o alcoolismo, a ausência de apoio social, assim como aspectos concernentes à maternidade, como fatores associados a déficits nutricionais na infância^1^ ^,^ ^26^. Alguns estudos sugerem que fatores presentes no âmbito do ambiente familiar poderiam afetar negativamente a capacidade materna de promover cuidados apropriados à criança, contribuindo para a desnutrição infantil^1^ ^,^ ^26^.

Ainda que crescente, a literatura que aborda as repercussões da saúde mental materna no estado nutricional infantil tem resultados contraditórios. Enquanto alguns estudos verificaram pior padrão de crescimento em crianças cujas mães apresentaram algum transtorno psicológico quando comparadas aos filhos de mulheres sem transtornos, outras investigações conduzidas em países em desenvolvimento não observaram diferenças significativas para esta relação^6^ ^,^ ^18^ ^,^ ^19^ ^,^ ^22^. Visando a esclarecer essa questão, o objetivo do presente estudo foi analisar se a saúde mental materna está associada ao estado nutricional infantil no sexto mês de vida.

## MÉTODOS

Este estudo faz parte de uma coorte prospectiva cujo objetivo central é investigar os determinantes sociais do processo de crescimento de crianças no primeiro ano de vida.

O presente estudo, de desenho seccional, tem como população fonte os recém-nascidos atendidos em quatro unidades básicas de saúde do município do Rio de Janeiro no momento da vacinação (BCG) ou do teste do pezinho, encaminhados diretamente das maternidades onde nasceram, conforme preconizado pelo programa “Acolhimento mãe-bebê”, da Secretaria Municipal de Saúde do Rio de Janeiro (SMS-RJ). As unidades básicas de saúde selecionadas foram escolhidas de forma intencional, levando-se em consideração o número médio de recém-nascidos que ingressam mensalmente no programa e a variabilidade geográfica, visando contemplar grupos populacionais diferentes.

As informações referentes à primeira entrevista (primeiro mês) e outra aos seis meses após o parto foram incluídas nesta pesquisa. Excluindo-se aquelas com anomalias congênitas, paralisia cerebral e seis gemelares, totalizou-se uma amostra de 228 crianças. Durante toda a coleta de dados, entre junho de 2005 e junho de 2008, a investigadora principal da pesquisa supervisionou os entrevistadores. Questionários com perguntas fechadas, pré-codificadas e outras abertas ou semiabertas foram aplicados em entrevista face a face com a mãe da criança.

Para obtenção das variáveis de desfecho que refletem o estado nutricional infantil foram aferidos peso e comprimento de crianças aos seis meses sem quaisquer vestimentas ou acessórios. O peso foi aferido em balança pediátrica da marca Filizola, com capacidade máxima de 15 kg (precisão de 0,1 kg). O comprimento foi aferido em antropômetro horizontal de madeira especialmente construído para a pesquisa (precisão de 1 mm). O treinamento antropométrico constou das seguintes etapas: (a) introdução sobre a avaliação nutricional de crianças menores de dois anos de idade; (b) apresentação teórica das técnicas de pesagem e medição de crianças menores de dois anos; e (c) padronização das mensurações antropométricas, procedimento recomendado por Habicht^5^ (1974). A partir dessas medidas, foram criadas as variáveis peso-para-comprimento e peso-para-idade segundo sexo, expressas em escores z, utilizando a nova curva de crescimento para menores de cinco anos^31^.

Para aferição da saúde mental materna seis meses após o parto, utilizou-se a versão validada e adaptada para o português do *General Health Questionnaire* com 12 itens (GHQ-12)^12^. Este instrumento tem como período de referência as duas semanas anteriores ao preenchimento do questionário e reflete sentimentos de depressão e ansiedade, incapacidade de lidar com situações habituais e falta de confiança^4^.

Cada item do GHQ-12 contém quatro opções de resposta, sendo que as duas primeiras consideram a experiência recente do participante em relação a um sintoma ou comportamento particular como ausentes (0 ponto atribuído) e as duas últimas como presentes (1 ponto atribuído). Assim, após o somatório dos pontos atribuídos às respostas de cada item, obtém-se uma escala de zero a 12 pontos potenciais. Foram estabelecidas três variáveis para avaliar a saúde mental materna a partir deste instrumento. A variável transtornos mentais comuns (TMC) foi definida a partir do ponto de corte de pelo menos três respostas positivas nos 12 potenciais. Esse ponto de corte apresenta boa sensibilidade (85,0%), sem prejuízo expressivo da especificidade (79,0%)^12^. Já a variável transtornos mentais mais graves foi identificada pelo ponto de corte de pelo menos cinco respostas positivas. Villano^a^ encontrou sensibilidade de 76,0% e especificidade de 59,0% para esse ponto de corte em um ambulatório de hospital universitário do Rio de Janeiro. Por fim, para a variável depressão, aplicou-se o ponto de corte de pelo menos nove itens positivos, conforme conduzido no estudo de Ozdemir e Remaki^17^, que encontraram sensibilidade de 75,0% e especificidade de 77,0%.

As covariáveis investigadas foram: idade materna (< ou ≥ 20 anos), trabalho materno (sim ou não), prematuridade (sim ou não), peso ao nascer (em gramas), número de filhos menores de cinco anos, escolaridade materna (anos completos de estudo) e rede social. Para avaliação da rede social, perguntou-se às participantes “com quantos amigos você se sente à vontade e pode falar sobre quase tudo?” e “com quantos parentes você se sente à vontade e pode falar sobre quase tudo?”. Em seguida, somou-se o número de parentes e amigos referidos para compor esta variável.

Inicialmente, estimaram-se as médias de escores z para os desfechos juntamente com as prevalências das variáveis de exposição, bem como as características sociodemográficas, maternas e da criança. As análises brutas foram conduzidas usando-se regressão linear simples (covariáveis categóricas e variáveis centrais de exposição) e teste de correlação de Spearman (covariáveis numéricas). Na análise multivariada, modelos de regressão linear foram ajustados pelas covariáveis que apresentaram associações com p ≤ 0,20 nas análises brutas.

Foram aplicados três métodos de diagnóstico dos modelos de regressão para avaliar a qualidade do ajuste: gráficos Q-Q plot, gráficos de resíduos de Student *versus* valores preditos e distância de Cook. Todos os modelos multivariados apresentaram bom ajuste e apenas um valor atípico foi encontrado, mas mantido nas análises.

Os dados foram armazenados no aplicativo EpiInfo, versão 6.04, e analisados nos pacotes estatísticos R, versão 2.9.0 e Stata, versão 9.2. O *software* WHO Anthro, versão 3.0.1, foi utilizado para construir os índices peso-para-comprimento e peso-para-idade.

O estudo foi aprovado pelo Comitê de Ética do Instituto de Medicina Social da Universidade do Estado do Rio de Janeiro e o ingresso à pesquisa se deu mediante assinatura pela mãe de um termo de consentimento livre e esclarecido.

## RESULTADOS

A Tabela 1 apresenta as características da população estudada. As crianças tinham idade média de 6,46 meses e 52,4% da amostra eram meninos. A minoria nasceu prematura e com baixo peso. Os escores z médios para os índices peso-para-comprimento e peso-para-idade foram, respectivamente, 0,23 e 0,05.

**Tabela 1:** Características da população de estudo. Rio de Janeiro, RJ, 2005-2008.

Variáveis categóricas	n*	%
Sexo da criança		
Masculino	122	52,4
Feminino	111	47,2
Peso ao nascer (gramas)		
Baixo (< 2.500)	16	6,9
Adequado (≥ 2.500)	216	93,1
Prematuridade		
Não	199	90,9
Sim	20	9,1
Trabalho materno		
Não	157	71,7
Sim	62	28,3
Idade materna (anos)		
≥ 20	173	77,2
< 20	51	22,8
TMC (GHQ-12)		
Não (< 3)	137	60,1
Sim (≥ 3)	91	39,9
Transtornos mais graves (GHQ-12)		
Não (< 5)	174	76,3
Sim (≥ 5)	54	23,7
Depressão (GHQ-12)		
Não (< 9)	209	91,7
Sim (≥ 9)	19	8,3

Variáveis numéricas	Média	DP

Peso-para-comprimento	0,23	1,08
Peso-para-idade	0,05	1,07
Peso ao nascer (gramas)	3,21	0,48
Idade da criança (meses)	6,46	0,80
Idade Materna (anos)	25,5	6,57
Escolaridade materna (anos)	8,49	2,81
Número de filhos < 5 anos	1,24	0,46
Rede (amigos e parentes)	3,67	2,87

* Totais podem variar devido à presença de valores faltantes em algumas variáveis.

As mães tinham, em média, 25,5 anos de idade, 8,5 anos de estudo, 1,24 filhos e 3,67 amigos ou parentes em quem podiam confiar para contar quase tudo (rede social). Um elevado percentual foi composto por mulheres adultas e que não trabalhavam na ocasião da entrevista. Aproximadamente 40,0% das mães foram detectadas com TMC, 24,0%, com transtornos mentais mais graves, e 8,0%, com depressão.

Nas análises brutas (Tabelas 2 e 3), apenas o peso ao nascer mostrou-se associado significativamente com os índices peso-para-comprimento e peso-para-idade, tendo sido a covariável de ajuste selecionada para ser incluída nos modelos de regressão linear múltipla.

**Tabela 2:** Análises bivariadas entre os índices peso-para-comprimento e peso-para-idade e as covariáveis categóricas (regressão linear simples). Rio de Janeiro, RJ, 2005-2008.

Variável	Peso-para-comprimento			Peso-para-idade		
	
	Média	DP	p	Média	DP	p
Prematuridade						
Não	0,21	1,09	-	0,05	1,06	-
Sim	0,18	1,02	0,906	-0,17	1,14	0,397
Trabalho materno						
Não	0,23	1,06	-	0,04	1,05	-
Sim	0,28	1,19	0,797	0,07	1,15	0,860
Idade materna (anos)						
≥ 20	0,21	1,09	-	0,06	1,07	-
< 20	0,26	1,08	0,766	-0,003	1,05	0,731

**Tabela 3:** Coeficientes de correlação de Spearman entre os índices peso-para-comprimento e peso-para-idade e as covariáveis numéricas. Rio de Janeiro, RJ, 2005-2008.

Variável	Peso-para-comprimento		Peso-para-idade	
	
	Coeficiente	p	Coeficiente	p
Peso ao nascer (gramas)	0,225	< 0,001	0,484	< 0,001
Escolaridade materna (anos)	-0,046	0,485	-0,010	0,875
Número de filhos < 5 anos	-0,001	0,990	-0,055	0,415
Rede (amigos e parentes)	-0,052	0,428	0,002	0,970

As associações brutas e ajustadas entre as variáveis de exposição e o estado nutricional das crianças estão apresentadas na Tabela 4. Os transtornos mentais mais graves e a depressão estiveram associados a menores escores z médios de peso-para-comprimento (Tabela 4). Os filhos de mulheres com transtornos mentais mais graves tinham, em média, 0,37 escore z mais baixo quando comparados às crianças cujas mães não apresentavam este agravo (p = 0,026). Os filhos de mulheres com depressão apresentaram média de 0,67 escore z mais baixo em relação aos filhos de mães não deprimidas (p = 0,010). Não se observou associação estatisticamente significante entre TMC e peso-para-comprimento.

**Tabela 4:** Coeficientes de regressão linear brutos e ajustados para a associação entre os índices peso-para-comprimento e peso-para-idade e as variáveis centrais de exposição. Rio de Janeiro, 2005-2008.

Variável	Peso-para-comprimento				Peso-para-idade			
	
	Bruto		Ajustado*		Bruto		Ajustado*	
			
	coeficiente	p	coeficiente	p	coeficiente	p	coeficiente	p
TMC (GHQ-12 ≥ 3)	-0,080	0,585	-0,053	0,714	0,022	0,882	0,048	0,708
Transtornos mais graves (GHQ-12 ≥ 5)	-0,393	0,019	-0,368	0,026	-0,025	0,884	-0,014	0,928
Depressão (GHQ-12 ≥ 9)	-0,674	0,011	-0,667	0,010	-0,474	0,065	-0,463	0,041

* Modelo ajustado pela variável peso ao nascer.

Ao analisar o índice peso-para-idade, crianças cujas mães foram detectadas com depressão apresentaram, em média, 0,46 escore z menor em relação ao grupo de filhos de mulheres sem depressão (p = 0,041). As outras variáveis de exposição não apresentaram associação estatisticamente significante com este desfecho (Tabela 4).

## DISCUSSÃO

Os resultados deste estudo mostraram que a saúde mental materna relacionou-se ao estado nutricional infantil aos seis meses de vida. Nos modelos ajustados foi encontrada associação entre menores escores z médios de peso-para-comprimento e as variáveis de exposição, exceto TMC. Para o escore z médio de peso-para-idade, a associação foi observada apenas com a depressão materna.

A literatura corrobora os achados deste estudo, pois vem indicando maior risco de desnutrição infantil nos primeiros seis meses de vida entre filhos de mulheres com depressão pós-parto^14^ ^,^ ^18^ ^,^ ^19^. Recente estudo longitudinal de Bangladesh encontrou a depressão entre dois e três meses pós-parto como fator de risco para déficit de peso entre seis e oito meses de vida, e a depressão gestacional, para déficit de comprimento-para-idade entre dois e três meses, e entre seis e oito meses de vida^14^.

Metanálise de 17 estudos sobre saúde mental materna e crescimento infantil verificou que há maior chance de déficits de peso e de comprimento-para-idade entre filhos de mulheres com depressão ou sintomas depressivos e ansiosos se comparados aos filhos de mulheres não deprimidas^27^. O efeito sobre esses déficits nutricionais teve maior magnitude nas análises que usaram o diagnóstico de depressão^27^. Os autores também encontraram maior efeito quando avaliaram os estudos longitudinais, porém entre esses a exposição investigada foi a depressão pós-parto e o resultado para déficit de comprimento-para-idade não foi significativo^27^.

No presente estudo se observou que o coeficiente da regressão aumenta conforme cresce a gravidade da exposição, isto é, a associação é mais forte entre os desfechos nutricionais com transtornos mais graves e, por conseguinte, com depressão. Como o gradiente biológico, ou efeito dose-resposta, é um critério relevante para inferir causalidade, os achados deste estudo, ainda que de natureza seccional, reforçam a hipótese de existência de efeito causal entre saúde mental materna e desnutrição infantil. Neste contexto, o efeito da exposição poderia ocorrer apenas a partir de um certo limiar que, no caso deste estudo, se daria a partir da presença de transtornos mentais mais graves. Nesse sentido, o nível de exposição no qual se classifica os TMC estaria abaixo do limiar de exposição, o que poderia justificar a não associação com os desfechos estudados.

Na literatura ainda não há consenso sobre a associação entre TMC materno e déficit nutricional. Enquanto alguns achados verificam relação positiva entre os eventos^6^ ^,^ ^15^ ^,^ ^21^ ^,^ ^22^, outros observam associações não significativas ou nulas^6^ ^,^ ^13^ ^,^ ^15^ ^,^ ^25^. É possível que a presença de TMC em um único momento não seja suficiente para modificar os desfechos relacionados ao estado nutricional.

Recente coorte prospectiva do Vietnã avaliou TMC entre mães no último trimestre gestacional ou entre quatro e seis semanas pós-parto (linha de base) e, novamente, aos 15 meses pós-parto, juntamente com o comprimento-para-idade. Segundo os autores, TMC na linha de base não se associou ao crescimento linear, mas houve maior risco de TMC materno aos 15 meses pós-parto entre mulheres com TMC perinatal e, ainda, a persistência de TMC perinatal e 15 meses pós-parto associou inversamente ao comprimento-para-idade aos 15 meses^3^.

Estudo caso-controle conduzido no Brasil encontrou cerca de duas vezes mais chance de déficit de peso-para-estatura em crianças com até seis anos cujas mães apresentassem TMC^22^. Em contrapartida, Stewart et al.^25^ não verificaram associação entre presença de TMC no pós-parto e menores escores z médios para o índice peso-para-idade em crianças aos nove meses. Medhin et al.^13^, em coorte da Etiópia com mais de mil participantes, não observaram efeito dos TMC no último trimestre gestacional, aos dois meses pós-parto, ou sua persistência entre esses dois momentos, sobre o déficit de peso ou o déficit de comprimento-para-idade aos seis e aos 12 meses de vida, nem mesmo na avaliação desses índices nutricionais de forma contínua.

Parte da heterogeneidade dos resultados entre os estudos pode ser explicada pela realização das investigações em momentos distintos do pós-parto ou ao método de mensuração dos transtornos mentais nessa fase. Adicionalmente, as divergências na literatura podem ocorrer devido a diferentes metodologias empregadas na escolha da variável de desfecho ou sua classificação ou ao uso de diferentes indicadores antropométricos. Em relação à classificação das variáveis de desfecho, por exemplo, alguns estudos aplicaram o ponto de corte de -2 escores z para detecção de déficit nutricional em crianças^6^ ^,^ ^15^ ^,^ ^22^, enquanto outros atribuíram percentis^18^ ou médias de escores z^21^ ^,^ ^25^. Diante disso, a comparação entre os achados é limitada.

Os resultados dos estudos também variaram de acordo com o indicador utilizado para avaliar prejuízos no crescimento infantil, bem como o país no qual o estudo foi conduzido^6^ ^,^ ^15^ ^,^ ^25^. Harpham et al.^6^ observaram associação entre TMC e déficit de comprimento-para-idade na Índia, mas não com déficit de peso-para-idade. O oposto ocorreu no Vietnã, com associação estatisticamente significante com o baixo peso-para-idade e sem associação com o comprimento-para-idade. Os autores não verificaram resultados significativos para Peru e Etiópia. Os mesmos resultados foram encontrados por Nguyen et al.^15^ ao avaliar as populações de Bangladesh, Vietnã e Etiópia. Segundo apontam alguns autores, as divergências nos resultados podem estar relacionadas com as diferenças culturais acerca das práticas alimentares e de cuidado com a criança entre os países estudados, bem como divergências socioeconômicas, tais como educação materna e segurança alimentar no domicílio^6^ ^,^ ^22^ ^,^ ^27^.

Os potenciais mecanismos que explicariam a relação entre saúde mental materna e crescimento infantil ainda não são claros. Conjectura-se que problemas na saúde mental materna podem prejudicar a habilidade da mãe em prover cuidados adequados à criança, por meio de práticas inadequadas de higiene, oferta insuficiente de alimentos e menor vínculo com o filho, o que poderia comprometer o crescimento infantil^1^. Mulheres com humor deprimido estão mais suscetíveis a ficar irritáveis e hostis com seus filhos e, nessas circunstâncias, podem negligenciar cuidados quando o bebê está com fome ou não consegue sugar adequadamente o leite materno, ou o controle higiênico sanitário na preparação da comida, o que pode levar à diarreia e, consequentemente, ao déficit nutricional^1^ ^,^ ^13^ ^,^ ^15^.

Já foi observado que sintomas depressivos no pós-parto representam maior risco para interrupção precoce do aleitamento materno, o que reflete a incapacidade materna de prover cuidado com a alimentação^7^ ^,^ ^19^. Por sua vez, a interrupção do aleitamento materno prejudica a evolução do ganho de peso^22^. Também foram relatadas menores diversidade na alimentação complementar, ingestão de energia, e frequência de refeições entre filhos de mulheres com sintomas depressivos e ansiosos^8^ ^,^ ^15^.

Outra via potencial para a relação entre saúde mental materna e desnutrição infantil poderia ocorrer pela prática inadequada de cuidados maternos com a saúde, tais como a prevenção de doenças e a busca por cuidado e serviços de saúde quando a criança está doente, levando ao adoecimento e à desnutrição infantil. Nguyen et al.^15^ mostraram que TMC materno está fortemente associado com diarreia e infecção respiratória aguda.

Apesar da hipótese sustentada de que os problemas psicológicos maternos possam induzir ao comprometimento do estado nutricional em crianças, ainda não há consenso sobre a temporalidade desta relação. O crescimento comprometido de crianças pode aumentar as preocupações maternas, fomentar sentimentos de inaptidão para o cuidado com a criança e levar ao incremento das pressões familiares, induzindo sentimentos de tensão e ansiedade, e mesmo a quadros depressivos mais graves^9^ ^,^ ^24^. Alguns estudos observaram que mães de crianças com problemas de saúde, baixo peso ao nascer e déficit de crescimento tinham maior risco de apresentar transtorno mental mais grave ou depressão no primeiro ano de vida da criança^9^ ^,^ ^20^ ^,^ ^27^.

As diferenças verificadas na literatura reforçam a necessidade de mais investigações de caráter longitudinal no primeiro ano após o parto. Também se faz necessária a condução de investigações que focalizem os possíveis mecanismos que podem interferir nessas relações.

Quanto à opção por utilizar o GHQ-12 para detecção de transtornos mentais mais graves e depressão, ainda que seja amplamente aplicado para rastreamento dos TMC na literatura, recentemente alguns estudos vêm utilizando este instrumento para aferição de transtornos mais severos e depressão^17^ ^,^ ^20^ ^,^ ^a^. Zubaran et al.^32^ verificaram correlação linear significante entre o GHQ-12 e as escalas largamente utilizadas para avaliar DPP (como a *Postpartum Depression Screening Scale* [PDSS] e a *Edinburgh Postnatal Depression Scale* [EPDS]), recomendando o uso de questionários de saúde geral para este agravo.

Nesta perspectiva, este estudo contribui para a discussão da determinação do processo de crescimento infantil e corrobora a hipótese de uma associação entre saúde mental materna e déficits nutricionais na infância.

Por fim, os resultados aqui expostos indicam a necessidade de incorporação da saúde mental em políticas e programas de atenção à saúde que incluam prevenção e tratamento de transtornos mentais, visando à melhoria da situação nutricional infantil. Em particular, recomenda-se que atenção mais cuidadosa seja oferecida na rotina dos serviços de saúde, tanto às mães de crianças com déficits nutricionais quanto aos filhos de mulheres com transtornos psicológicos. O uso de ferramentas acessíveis nos serviços de saúde para o reconhecimento de problemas psicológicos após o parto vem sendo recomendado^b^. Uma abordagem simples seria a inclusão de questionários de rastreamento, como o GHQ-12, e capacitação das equipes, com o objetivo de ampliar a detecção dessa população nas rotinas de atendimento.

## Footnotes

a Villano LA. Problemas psicológicos e morbidade psiquiátrica em serviços de saúde não-psiquiátricos: o ambulatório de clínica geral [tese]. São Paulo (SP): Universidade Federal de São Paulo; 1998.

b World Health Organization, Department of Mental Health and Substance Abuse. Maternal mental health and child health and development in low and middle income countries: report of the meeting held in Geneva, Switzerland; 2008 30 Jan-Feb 2008. Geneva: World Health Organization; 2008.
